# Fitting a Cap or a Band to a Root

**Published:** 1892-06

**Authors:** 


ARTICLE V.

### FITTING A CAP OR A BAND TO A ROOT.

Dr. Charles J. Essig: The operation of fitting a cap or a ring to a root is very difficult. I have seen several roots that have been removed after bridges or crowns have been fastened to them, and I have rarely seen one where there has been precision in fitting the band. The form of the root is often very tapering, and in fitting a band under the gum we are apt to fail, through difficulty in determining its exact form, to obtain a perfect adaptation of the ferrule to the root. Where I have had the chance to prepare a root to my own liking, I spare as much of the tooth as possible, and do not go very far beyond the margin of the gum. Suppose we have a central tooth which is discolored or broken to such an extent that we find it necessary to crown it. How shall we prepare that root? Shall we cut it off in its entire circumference to the margin of the gum? I should cut the root off so as to save as much as possible of the lingual portion and beveling to a little above the margin of the gum on the outer or labial portion, so that the porcelain face may be carried under the gum to conceal the line of union. No gold should be visible in front. The cap I prefer is a partial cap.

There is another important point, the manner of measuring the root and fitting the cap. It seems to me that a plaster model would be much better to depend on than a piece of wood which is made hap-hazard, or a piece of wire fitted to the root. Measuring by wire coiled around the root is largely employed, but has never been satisfactory to me. After we have gotten such a model as plaster will give us, we certainly have a pretty good fac-smile of our case from which we may obtain very reliable measurement. The next step is to cut away the plaster model below the margin of the gum; so as to allow you to fit the cap. This model will be found to be pretty nearly like the root. If it is made of good plaster and thoroughly dried, then well saturated with thin sandaric varnish, and allowed to dry again, you will have a model that is hard enough to work on without injury; but precision in fitting the cap can, in many cases, only be obtained by fitting the band as well as you can, on your model; soldering it together, or making the ring first before you put the floor on, and then fitting it on the root itself. Ferce it up into the position that you want it in, aad then see how it fits. Of course, it is well at the very beginning to make some preliminary preparation of the root. If you can determine that the edge of the root which is out of the gum has a decidedly tapering form, this must be remedied before taking the impression.

There must be precision in fitting the cap and pin together. Without the cap, the pin sustains all the force. The force of mastication is thrown on the long axis of the tooth instead of on the socket, where it properly belongs, and there is danger of fracture of the root of the tooth; we have all doubtless met with such accidents. If the cap does not fit the root well, it is no reinforcement to the pin; so it becomes necessary, to make one serve the other, to have the cap fit accurately. Much of the work of preparing crowns may be done by the mechanical dentist, and this is a saving to both operator and patient. I do not like to make a laboratory of my office, and a method of procedure which obliges a dentist to go from the work-bench to the mouth of the patient will prove wearing and distasteful to both. The plaster model, on which most of the work can be accomplished, will save much time to the dentist and annoyance to the patient.

After the ring or ferrule has been so made, however, it may then be adjusted to the natural root. When the cap is fitted on the root, if it stands away from the sides, it can be burnished back; it cannot be adjusted to the root accurately at any other time. Then you can finish putting the floor on, and the cap is then ready for the reception of the tooth; but it would be better to try it once more in the mouth. Dr. Case remarked about the accuracy of the adjustment of the pin. The correct relation of the pin and cap is important. If the pin fits well and the cap stands away from the root, it will give no additional strength to the pin, and, when the force is thrown on it, it may bend or break, or the root may split.

I have found pure gold too ductile, so that even after we have fitted a cap to a tooth, with the object of making it reinforce the pin, it is quite possible that we may have a yielding of the soft gold. I much prefer one of the alloys of gold. Coin or standard gold affords greater strength and better results. Coin gold is very much stronger; it has a tendency of 70 or 80, while pure gold has only about 20 or 25, and I therefore favor the use of coin gold. By taking proper precaution and giving every detail of the operation proper care, we can get a sufficient adjustment, without depending on the burnisher to make the cap fit the root.

Dr. F. Van Woert: It is very strange, nevertheless a fact, that bicuspids and molars take kindly to bands or ferrules, while the incisors and cuspids are the reverse, especially centrals. Therefore I never use a band crown on these teeth, unless absolutely necessary. The only possible advantage of a band is to add strength to the crown and root, or, in other words, protect the root from fracture. This same object may be accomplished in a different manner without encroaching on the pericemental membrane, as follows:

In the preparation of a root, the face should be cut on a straight line from the center to the gum-margin on the labial surface, forming an incline; and from the center to palatial gum-margin, forming a quarter-circle. Then drill, and fit the dowel into the root; after which bend a piece of pure gold the shape of the root as nearly as possible; trim to something like the contour of the circumference, after which place it on the end of the root, and drive the pin through it and to place. Now remove the whole, solder, and adjust, when the plate can be perfectly adapted to the ends of the root by a small pine stick and a gold plugging mallet. Now, by removing, you will find the outside margins of the root clearly defined on the under side of the plate, permitting you to follow accurately with the corundum wheel the exact shape of the root. After soldering the face to this floor, you have a crown possessing all the advantages of a band crown, and the great merit of not encroaching on the pericemental membrane.

I am surprised to learn that there are members of our profession who so enjoy labor that they attempt to finish bands after a crown has been set. They certainly cannot believe it possible to finish a band in this manner, anything like perfectly; in fact, I have great doubts as to whether it is possible to half accomplish this feat without permanent injury to the root. By all means, finish the band before setting the crown.

Roots should be conically shaped, so that when a band is driven on it will stretch to a close union at the edge. There are frequently small inequalities which it is better to burnish the band to than to attempt to cut away; but it is less difficult to burnish to one or two small defects of this kind than it is to the whole circumference of the root.

I do not believe it is better to work from a model. I

prefer the natural root. As to the instruments used for trimming roots, I have never been able to secure one that would accomplish what corundum wheels and emery-paper discs will do. I prefer them to any steel instrument that I have ever seen.

This principle of crowning preserves very much more of the tooth-substance than any other I know of. The principle of the V and half-circle, or a combination of both, in the preparation of a root to receive a crown, is an old one; but I consider it one of the best. I am opposed to the least sacrifice of tooth-substance where it can possibly be saved.

Respecting banding crowns, I have cut off the crowns of a badly-decayed superior left first bicuspid, shaped the root conically, finished and adjusted the band, applied the rubber-dam, filled the roots, and inserted a porcelain tip; the entire operation consuming thirty-eight minutes. The complication of any operation lessens the chance of its final success, and certainly adds very much to the discomfort of the patient and operator, to say nothing of its being less profitable to the dentist.

Dr. C. Frank Bliven: In constructing artificial crowns there are three things to be considered—strength, beauty and ease of repair.

Strength is the most essential quality. Take an incisor. You are all familiar with its anatomical construction, and the manner in which the enamel overlaps the dentine. The nearer we conform to these natural principles, the stronger the crown will be. In the selection of a material, I believe we have in porcelain the only material at the present day that answers the purpose. In preparing the borders, the more we cut the tooth away toward the apex, the weaker the crown; for instance, in the Richmond crown the force is applied in an oblique angle, making a fulcrum at the labial point of attachment, thereby having a tendency to lift the pivot from the root, as a tack or nail is drawn with a claw-hammer.

Dr. Van Woert's method, which he has just described, seems to me to be much more practical, because it brings the fulcrum nearest to the point of greatest resistance.

If a cap must be saved, use the band itself to take the measurement, place it around the root with a ligature about it, draw it tight, cut and solder. This, to me, seems the most practical method. The taking of an accurately fitting cap is one of the most difficult things we have to do.

But, with few exceptions, I find it unnecessary to make caps. The burnishing of a piece of pure gold onto a hickory stick, removing it and having it fit the root accurately as a cap should, is almost an impossibility.

Dr. Baker, of Boston, several years age introduced a method which was a good one, if we do not object to the extra labor. He places a piece of thin copper about the root (tagger's tin is best for this purpose), ligates it to hold it securely in place, and forces plaster of Paris into the open end; after the plaster has set, it is removed, and fusible metal is poured into the end that was about the root. This makes an excellent model on which to construct the cap.

The next point to consider is that of beauty. All methods where the band must show are undesirable. For this reason I strongly object to them, and I rarely find them necessary. There are other simpler and superior methods. Prepare the root like a truncated cone, concaving the labial surface; prepare a platina cornicopia, introduce the pivot at the apex, trimming the platina form so that it will extend slightly under the margin of the gum, and burnish to place. On this form construct an all porcelain crown similar to thickened enamel. For strength, beauty and ease of repair, this method, in my practice, has been prolific of the best results. This crown gives me a border at once strong, beautiful and non-irritating—one that is not approached by any other method with which I am familar.

Dr. S. C. G. Watkins: This subject is one of great interest to all of us. Dr. Van Woert spoke of the fitting of bands, and the inference would be drawn from his remarks that it was a very common thing to see bands well fitted, and an uncommon thing to see poor work. It seems to me that the majority of bands are fitted miserably. We see much of carelessness, slovenly work in crown and bridge-work. Either men ought not to attempt it, or qualify themselves specially for it. Dr. Case refers to the cutting down of teeth only half-way, and leaving free surfaces which are easily cleansed. That I think, is an excellent idea. When a sound molar, for instance, is trimmed for attaching a crown to it, Dr. Case's idea is to trim the tooth half way to its neck, so as to get a solid bearing. Then fit the cap on that, and burnish the gold band to make a close joint. It seems to me to be a more practical method than covering the entire tooth with gold, and taking the chances of having an imperfectly fitting band under the edge of the gum.

I do not believe in banding in crown-work if it can be avoided. I think a tooth which is crowned without a band is sufficiently strong, and less liable to cause after trouble, providing the root is good, and not cut away too much in its preparation.

The tendency in prosthetic dentistry is toward the use, wherever possible, of artificial substitutes which do not cover the roof of the mouth, as in bridge-work. Where the bridge-work is removable, the patient can keep it thoroughly cleansed. I have been experimenting with much satisfaction, doing away with the covering of the roof of the mouth in pieces containing from one to five or six teeth, by the use of a spring plate. Where, for instance, the second bicuspid is badly decayed, with the first molar on the same side decayed on the two proximal surfaces, and the second molar also decayed on the anterior proximal surface, I have adopted the plan of cutting away the crown of the first molar, which gives perfect access to the cavities in the other two teeth, which are then filled and a crown placed on the tooth which was cut away. My idea is that the operation can be performed in less time, the fillings can be inserted more accurately, and the crown be a more certain protection for the first molar, than the large compound filling which would be necessary.—*Items of Interest.*

